# Modulation of the Wheat Seed-Borne Bacterial Community by *Herbaspirillum seropedicae* RAM10 and Its Potential Effects for Tryptophan Metabolism in the Root Endosphere

**DOI:** 10.3389/fmicb.2021.792921

**Published:** 2021-12-23

**Authors:** Pablo Carril, Joana Cruz, Claudia di Serio, Giuseppe Pieraccini, Sylia Ait Bessai, Rogério Tenreiro, Cristina Cruz

**Affiliations:** ^1^Plant-Soil Ecology Laboratory, Faculty of Sciences, Center for Ecology, Evolution and Environmental Changes (cE3c), University of Lisbon, Lisbon, Portugal; ^2^Geriatric Intensive Care Unit, Experimental and Clinical Medicine Department, University of Florence, Azienda Ospedaliera Universitaria (AOU) Careggi, Florence, Italy; ^3^Department of Health Sciences, Mass Spectrometry Centre (CISM), University of Florence, Florence, Italy; ^4^Laboratoire de Maîtrise des Énergies Renouvelables (LMER), Faculté des Sciences de la nature et de la vie, Université de Bejaia, Bejaia, Algérie; ^5^Faculty of Sciences, BioISI – Biosystems and Integrative Sciences Institute, University of Lisbon, Lisbon, Portugal

**Keywords:** plant microbiota, seed-borne endophytic bacteria, microbial inoculants, root endosphere, tryptophan metabolism, wheat

## Abstract

Plants and their associated microbiota share ecological and evolutionary traits that are considered to be inseparably woven. Their coexistence foresees the use of similar metabolic pathways, leading to the generation of molecules that can cross-regulate each other’s metabolism and ultimately influence plant phenotype. However, the extent to which the microbiota contributes to the overall plant metabolic landscape remains largely unexplored. Due to their early presence in the seed, seed-borne endophytic bacteria can intimately colonize the plant’s endosphere while conferring a series of phytobeneficial services to their host. Understanding the dynamics of these endophytic communities is a crucial step toward the formulation of microbial inoculants that can modulate the functionality of the plant-associated microbiota for improved plant fitness. In this work, wheat (*Triticum aestivum*) roots non-inoculated and inoculated with the bacterium *Herbaspirillum seropedicae* strain RAM10 were analyzed to explore the impact of inoculant–endophyte–wheat interrelationships on the regulation of tryptophan (Trp) metabolism in the endosphere environment. Root inoculation with *H. seropedicae* led to phylum-specific changes in the cultivable seed-borne endophytic community. This modulation shifted the metabolic potential of the community in light of its capacity to modulate the levels of key Trp-related metabolites involved in both indole-3-acetic acid (IAA) biosynthesis and in the kynurenine pathway. Our results support a mode of action of *H. seropedicae* relying on a shift in both the composition and functionality of the seed-borne endophytic community, which may govern important processes such as root growth. We finally provide a conceptual framework illustrating that interactions among roots, inoculants, and seed-borne endophytes are critical to fine-tuning the levels of IAA in the endosphere. Understanding the outcomes of these interactions is a crucial step toward the formulation of microbial inoculants based on their joint action with seed-borne endophytic communities to promote crop growth and health in a sustainable manner.

## Introduction

Plant–microbe beneficial associations have been the focus of intensive research, given their potential as sustainable alternatives for agricultural production (reviewed in Gupta et al., 2021). In this context, the transition of seeds to seedlings represents one of the most critical stages of plant development, where microbial interactions taking place in these early stages can profoundly impact host ecology, health, and productivity (Nelson, 2018). The seed endophytic habitat represents a protected environment, conferring an ecological advantage to the microbiota inhabiting it to interact closely with their host and influence plant phenotype. Seed-borne endophytic bacteria (from now on referred to as seed-borne endophytes) are a key class of plant symbionts associated with the plant throughout, at least, part of its life history, without causing negative impacts on their host (Guo et al., 2021). Due to their early and intimate niche occupation, seed-borne endophytes represent a starting point for community assembly in the new seedling and are considered central players in determining the trajectories for crop success in agricultural systems (Shahzad et al., 2018; Taulé et al., 2021). Recent advances in plant-microbiome engineering have opened new perspectives for the promotion of plant health by exploiting the potential of seed-borne endophytes (Guo et al., 2021). In this context, understanding their contribution to plant phenotype is a crucial step for the design of promising microbial inoculants to improve plant growth and health through the manipulation of the microbiota functionality (Truyens et al., 2015; reviewed in Berg et al., 2021). Despite the beneficial effects of seed-borne endophytes, their metabolic contribution to plant phenotype and especially their functional modulation by bacterial inoculants remain largely unexplored.

The plant’s metabolic status is dictated by the combined action of the plant’s own regulatory pathways and by the activities of its associated microbiota. Bacterial endophytes possess the ability to produce or degrade phytohormones, with most data accumulated for the production of metabolites that intervene in the indole-3-acetic acid (IAA) synthetic pathway from tryptophan (Trp; Alkhalaf and Ryan, 2015; Gaetani et al., 2020). Both plants and microbes can utilize Trp through shared or dissimilar catabolic pathways, contributing to the generation of different metabolites that can cross-regulate each other’s metabolism (Costantini et al., 2020). For example, both partners share several metabolites involved in four different IAA biosynthetic pathways: indole-3-pyruvate, tryptamine (Try), indole-3-acetonitrile, and indole-3-acetamide (IAM) pathways. Fine-tuning of IAA levels in plants is essential to promote root development, among other key plant developmental processes (Di et al., 2016). Other Trp catabolic processes, such as 5-hydroxytryptamine (5-HT) synthesis through the Try pathway, have primary roles in plant resistance against stress and root growth (Schillaci et al., 2021). Despite the uncertainty of microbial synthesis of 5-HT, bacteria can produce metabolites influencing 5-HT levels in the host (Yano et al., 2015). Bacteria can also modulate Trp availability through the regulation of the kynurenine (Kyn) pathway. In this regard, the evolutionary conservation of the Kyn pathway in mammals and bacteria is important for the synthesis of nicotinamide adenine dinucleotide (Costantini et al., 2020). Although there are little data on the Kyn pathway in plants, its potential role as a suppressant of auxins and modulator of root growth has been postulated in *Arabidopsis thaliana* (He et al., 2011). Another Trp-derived metabolite, indole-3-carboxaldehyde (I3A), can be produced by probiotics such as lactobacilli (Zelante et al., 2013). However, while having important immunomodulatory functions in humans, its microbial production in plants has never been tested.

Considering the similarities in the catabolism of Trp shared by plants and bacteria, the “plant–inoculant–endophyte” tripartite system represents an excellent model to study the contribution of both inoculants and seed-borne endophytes to the modulation of Trp metabolism in plants and to predict its consequences for plant phenotype. In contrast to the increasing number of studies in the rhizosphere environment, very little is known regarding the contribution of inoculants and seed-borne endophytes to the endosphere’s Trp metabolic landscape.

*Triticum aestivum* (wheat) is one of the most cultivated crops worldwide. However, its production relies heavily on agrochemicals, such as fertilizers and pesticides, which can be environmentally harmful due to their excessive use (Kavamura et al., 2021). During the last decades, seed-borne endophytes from several wheat varieties have been isolated, characterized, and proposed as potential alternatives for plant-growth promotion (Kuźniar et al., 2020). The β-proteobacterium *Herbaspirillum seropedicae* is a beneficial bacterium isolated from various cereal plants (Olivares et al., 1996), which has the potential for an intimate root association and growth promotion in different cereals. However, whether the mode of action of *H. seropedicae* relies on the modulation of the seed-borne endophytic community composition and functionality remains unexplored. In this work, wheat plants inoculated with *H. seropedicae* were selected as a model system to study inoculant–endophyte–wheat interrelationships. First, the diversity, structure, and persistence of the cultivable seed-borne endophytic communities extracted from non-inoculated and *H. seropedicae*-inoculated roots were characterized. Secondly, the optimization of a methodology to grow these communities in root extracts, together with metabolomic analyses, allowed to explore the metabolic potential of seed-borne endophytes in light of their capacity to modulate the levels of key Trp-related metabolites in the root endosphere. The present study contributes to the understanding of the crosstalk between plants, seed-borne endophytes, and inoculants and explores an overlooked mechanism of action of inoculants—the modulation of the seed-borne endophytic community composition and functionality.

## Materials and Methods

### *Herbaspirillum seropedicae* Growth Conditions

*H. seropedicae* strain RAM10, isolated from *Graminaceae* plants (Olivares et al., 1996), was grown in LB medium [tryptone, 10 g/L; yeast extract, 5 g/L; sodium chloride (NaCl), 10 g/L; pH 7.0] and incubated for 24 h in a rotary shaker (28°C, 120 rpm), centrifuged (2,374 × *g*, 10 min, 15 min), and resuspended in 25 ml of 1/4 diluted Hoagland solution (Hoagland and Arnon, 1950) to an optical density at a wavelength of 600 nm (OD_600_) = 1 (10^9^CFU/ml).

### Plant Growth Conditions and Treatments

Wheat (*T. aestivum*, variety Jordão) seeds were surface-sterilized (1.5 min in 70% ethanol; 1 wash in sterile deionized water (s.d.w.), 3 min in sodium hypochlorite; 10 washes in s.d.w.), soaked for 12 h in s.d.w. and heat-treated (10 min, 50°C). Seeds were then transferred aseptically to Petri dishes containing 1.5% water agar (10 seeds per dish) and kept in a growing chamber with a 16-h light/8-h dark photoperiod and temperature of 25/20°C (RH = 70/50%), for 96 h. Four-day-old seedlings were transferred to tip boxes containing 250 ml of 1/4 diluted Hoagland solution, with the leaves emerging from the holes of the rack and the solution bathing the roots. Twenty-one seedlings per box were transferred. A total of 12 boxes were prepared, half of which were kept as control (referred to as control roots), and the other half were root-inoculated with *H. seropedicae* (referred to as inoculated roots) to a final density of 10^8^ CFU/ml immediately after seedlings transfer to the boxes. Each box was sealed in sterilized gas exchange bags and maintained in the growth chamber for 2 weeks. Six boxes (three *H. seropedicae*-inoculated and three non-inoculated) were used for the isolation of cultivable seed-borne endophytes from the roots and the other six (three *H. seropedicae*-inoculated and three non-inoculated) for the preparation of root extracts and LC-MS analyses.

### Modulation of the Seed-Borne Endophytic Community by *Herbaspirillum seropedicae*

#### Isolation and Characterization of Seed-Borne Endophytes From Control and Inoculated Roots

The first goal of this study was to assess whether the inoculation of *H. seropedicae* induced changes in the composition of the cultivable seed-borne endophytic community present in wheat roots. Bacteria were isolated from roots after 9, 16, and 21 days after germination (d.a.g.). At each time point, a pool of seven roots was aseptically collected from each biological replicate, weighted and added to 30 ml s.d.w. This root suspension was macerated with mortar and pestle and kept at room temperature for 15 min. Suspensions were serially diluted, 10 μl of each dilution dropped in LB Petri dishes of 9 cm diameter and incubated overnight (28°C, 120 rpm). Dilutions containing between 30 and 300 colonies were replated (50 μl) in new LB agar plates and incubated for 48 h in the same conditions. Colonies were selected on the basis of their morphologies, and unique morphotypes were characterized through Gram, KOH, catalase, and oxidase tests (Cappuccino and Sherman, 2004; list of isolates and phenotypic characterization, Supplementary Table 1) and subjected to polymerase chain reaction (PCR) fingerprinting for bacterial strain-specific discrimination, where the M13 core sequence was used as a single primer. Each PCR mixture contained 2.5 μl of 10 × buffer, 0.5-μl M13 primer (50 pmol/μl; sequence: 5′-GAGGGTGGCCGGTTCT-3′), 0.5-μl deoxynucleoside triphosphate mix (10 mM), 1.5-μl magnesium chloride (50 mM), 0.2-μl Taq DNA polymerase (5 U/μl), 5-μl DNA and s.d.w. to 20 μl (amplification: initial denaturation at 95°C—5 min, followed by 40 cycles of 95°C—1 min, annealing at 50°C—2 min, extension at 72°C—2 min, and a final extension at 72°C—5 min). PCR products were visualized by electrophoresis in 1.2% (w/v) agarose gel (running time: 3 h, 80 V) after ethidium bromide staining (Sambrook et al., 1989). Fingerprinting profiles were compared using BioNumerics software (Version 5.1, Applied Maths NV).

Bacterial DNA was extracted by resuspending each bacterial morphotype in saline solution (0.8 NaCl) and subsequent heat-treatment (70°C, 10 min) in a heat block. Molecular identification was carried out through amplification and sequencing of the partial 16S recombinant DNA (Eurofins, SupremeTube-Supreme Sanger Sequencing) region using universal primers 104F and 1392R. Each PCR mixture contained 5-μl buffer (10×), 1 μl of 104F primer (50 pmol/μl; sequence: 5′-GGACGGGTGAGTAACACGTG-3′), 1392R primer (50 pmol/μl; sequence: 5′-ACGGGCGGTGTGTRC-3′), 1-μl deoxynucleoside triphosphates mix (10 mM), 2-μl magnesium chloride (50 mM), 1-μl Taq polymerase (5 U/μl), 2-μl DNA and s.d.w. to 50 μl (amplification: initial denaturation at 94°C—3 min, followed by 35 cycles of denaturation at 94°C—1 min, annealing at 55°C—1 min, extension at 72°C—1 min, and a final extension at 72°C—3 min). Sequencing products were identified using the BLAST tool of the National Center for Biotechnology Information. Within each cultivable seed-borne endophytic community isolated from control or inoculated roots, persistent strains were defined as those found in all root developmental stages, whereas transient strains were defined as those having fluctuations in their presence based on their detectability thresholds in LB plates.

#### Antagonistic Relationships Among Seed-Borne Endophytes and Between Seed-Borne Endophytes and *Herbaspirillum seropedicae*

Growth inhibition activity was tested both between all the members of the seed-borne endophytic community isolated from control roots against those isolated from inoculated roots and between the members of both seed-borne endophytic communities against the inoculant *H. seropedicae*, using a modified cross-streak method (Velho-Pereira and Kamat, 2011). Petri dishes containing solid LB medium (1.5% agar) were prepared and inoculated with the antagonist by a single streak in one side of the Petri dish and incubated at 28°C for 3 days. Then, plates were seeded with test organisms by streaking perpendicular to the line of antagonist growth. Plates were incubated again at 28°C for 3 days. For each isolate, this experiment was repeated three times.

### Bacterial-Mediated Modulation of Tryptophan Metabolism

#### Experimental Strategy for High-Performance Liquid Chromatography–Tandem Mass Spectrometry Analyses

In the second part of this study, the effects of *H. seropedicae* inoculation on the levels of several Trp-related metabolites in the roots was determined. Then, the modulation of the levels of these metabolites by the seed-borne endophytic community isolated from control or inoculated roots was evaluated. For this, control and inoculated roots were collected, weighted, frozen in liquid N_2_, and homogenized with mortar and pestle. Then, 100 mg of root powder were resuspended in 1 ml of 80% methanol (MeOH), kept at 70°C for 5 min, and centrifuged two times (10 min, 13,450 × *g*). The supernatant was transferred to new tubes, evaporated to dryness at 45°C using a rotary vacuum evaporator (Jouan, Thermo Fisher Scientific), resuspended in 100 μl of MeOH 80% (1 mg root/μl) and kept at −20°C until liquid chromatography–mass spectrometry (LC-MS) analyses. The experiment was performed in triplicate. Individual members of both seed-borne endophytic communities were grown overnight in LB medium, centrifuged, resuspended in M9, and their optical density adjusted to 0.4. Members belonging to the community isolated either from control or inoculated roots were mixed at a 1:1 ratio (v/v) in two different tubes. A 20-μl aliquot of the mix containing the community extracted from control roots was transferred to 96-well microplates containing 180 μl of minimal medium M9 (monopotassium phosphate, 3 g/L; NaCl, 0.5 g/L; disodium hydrogen phosphate, 6.5 g/L; ammonium chloride, 1 g/L; glucose, 1 g/L) supplemented with extracts of either control or inoculated roots (final concentration: 1 mg root/μl). The same was done with the mix containing the community extracted from inoculated roots. Microplates were incubated overnight (28°C, 180 rpm), and cultures were filtered using a 22-μm syringe filter (DynaGard). Filtered supernatants were vacuum evaporated to dryness at 45°C and kept at −20°C until LC-MS analyses. The experiment was performed in triplicate.

In a parallel assay with the same experimental setup, the growth of both seed-borne endophytic communities in M9 supplemented with the extracts of either control or inoculated roots was monitored by measuring OD values (OD_600_) every 6 h during 24 h using a Tecan infinite M200 microplate reader (Tecan Austria GmbH). The growth stimulatory effect of root extracts was calculated as the fold-change increase in OD values of each seed-borne endophytic community in M9 supplemented with root extracts relative to OD values in M9 without extracts.

#### Chemicals and Reagents for High-Performance Liquid Chromatography–Tandem Mass Spectrometry Analyses

All solvents and reagents were LC-MS grade and were supplied from Sigma-Aldrich (Merck, Milan, Italy). Individual stock solutions of both isotopically labeled internal standards and unlabeled analytes were prepared in MeOH at 1.0 mg/ml and stored at −80°C. The labeled internal standards were *l*- Kyn sulfate (ring-d4,3,3-d2, 97% +) 95% (Cambridge Isotope Laboratories, CIL, United States); *dl*-3-hydroxykynurenine:HCl (^13^C_2_, 99%; ^15^N, 98%, CIL); indole-2,4,5,6,7-d5-3-acetic-2,2-d2 acid (99.2%, CDN Isotope, Point-Claire, Quebec, Canada); anthranilic-3,4,5,6-d4 acid (98%, CDN). The chemical standards were *l*-Kyn, 3-hydroxy-*dl*-Kyn, IAA, anthranilic acid 99.5%; 3-hydroxyanthranilic acid 97%; indole-3-acetamide 98%; indole-3-carboxaldehyde 97%; indole-3-acetaldehyde 98%; indole-3-acetonitrile 98%; 5-HT 98%; tryptamine; tryptophan. Two separate working standard solutions, one containing all the isotope-labeled standards and one containing all the unlabeled standards, were prepared at 2.5 and 10 ng/μl, respectively, diluted in water with 5-mM ammonium acetate and 0.2% formic acid (eluent A) and stored at −20°C.

In this study, *l*-Kyn-d6 was used as labeled internal standard for Kyn, tryptophan, tryptamine, and 5-HT, whereas IAA d7 was used as an internal standard for all the indoles measured in this study. Internal standards were used as tracer molecules to adequately compare all the different samples and not for quantitative analyses in the strict sense. For this, internal standards were always added in the same quantity to all samples before LC-MS analyses to verify the reproducibility and yield of the extraction procedure, to check for the matrix effect in electrospray ionization process, and to finally normalize the different signal intensities for comparison between samples.

#### Liquid Chromatography–Mass Spectrometry Analyses of Tryptophan Metabolites

For the analysis of Trp metabolites, the extracts of both control and inoculated roots and the cultures containing the isolated seed-borne endophytic communities grown in the extracts of either control or inoculated roots were resuspended in 80-μl MeOH and 20 μl of internal standard solution and diluted 1:1 with eluent A. All samples were sonicated (5 min) and centrifuged (13,450 × *g*). The supernatant was then transferred in an autosampler vial and injected into the high-performance liquid chromatography–tandem mass spectrometry instrument. A Series 200 (Perkin Elmer) high-performance liquid chromatography system was used coupled to a 4,000 Qtrap hybrid triple quadrupole LC-MS/MS (Sciex) equipped with a TurboV Ion Spray source operating in positive ion mode. Analyst software (v.1.6.2) from Sciex was used for data acquisition and analysis. All MS parameters were optimized by direct infusion and source parameters (gas flows and temperatures) by flow injection. The ion source operated with ion spray voltage set at 5.3 kV, curtain gas at 24, ion source temperature at 550°C, and ion source gas GS 1 and GS 2 were at 70 and 48 ml/min, respectively; collision gas was nitrogen at 3.4 × 10^–5^ bar pressure. Analytes were detected using scheduled multiple reaction monitoring acquisitions; two transitions were monitored for each molecule. All the acquisition parameters are listed in Supplementary Table 2. An Ultra AQ C18 column (100 × 2.1 mm, 3 μm; Restek, United States) was used; eluents were 5-mM ammonium acetate in water (A) and acetonitrile (B), both containing 0.2% formic acid. The column temperature was kept at 35°C. Chromatographic separation of the analytes was performed using a linear gradient as reported in Supplementary Table 3; 10-μl injection volume was used. The column effluent was delivered to the mass spectrometer with no split. Peak area ratio (PAR) values for each Trp analyte present in the extracts of both control and inoculated roots and in the cultures containing each bacterial community grown in the extracts of either control or inoculated roots were calculated by dividing the area of the main transition of each analyte peak relative to that of the main transition of its corresponding internal standard. The percentage of increase/decrease of PAR values in the cultures was then calculated relative to the PAR values of the extracts of control or inoculated roots.

## Results

### *Herbaspirillum seropedicae*-Induced Modulation of Seed-Borne Endophytic Community

**FIGURE 1:**
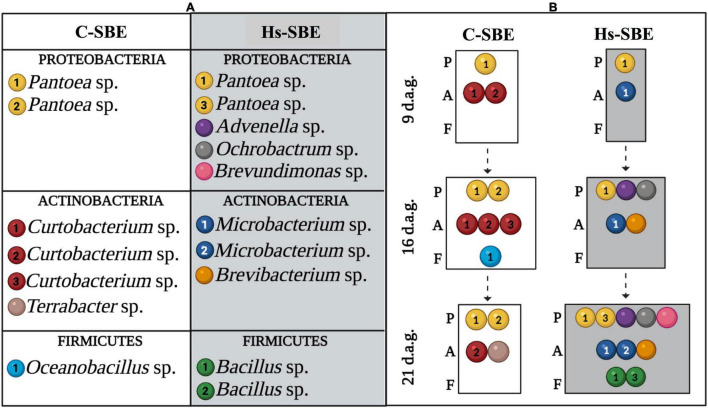
**(A)** Representative bacterial strains belonging to seed-borne endophytic community isolated from either control or inoculated roots (white and gray background, respectively) and identified based on PCR fingerprinting profiles and 16S-based identification. Colors of circles represent different genera, and different numbers correspond to different strains belonging to same genus. **(B)** Representative strains from control or inoculated roots (white and gray background, respectively) identified throughout root development. C-SBE, seed-borne endophytes isolated form control roots; Hs-SBE, seed-borne endophytes isolated form inoculated roots; d.a.g., days after germination; P, *Proteobacteria*; A, *Actinobacteria*; F, *Firmicutes*. Created with BioRender.com.

To assess whether root inoculation with *H. seropedicae* strain RAM10 could shape the composition of the seed-borne endophytic community, both control and inoculated roots were analyzed to characterize their cultivable bacterial communities. The members of both communities belonged to the phyla Proteobacteria, Actinobacteria, and Firmicutes (Figure 1A), and both communities harbored persistent and transient strains (Figure 1B). Strains of the seed-borne endophytic community isolated from control roots belonged to the genera *Pantoea* (Proteobacteria), *Curtobacterium* and *Terrabacter* (Actinobacteria), and *Oceanobacillus* (Firmicutes). Here, one strain from *Pantoea* and another from *Curtobacterium* were found as persistent endophytes, although these genera also harbored transient strains. The genera *Terrabacter* and *Oceanobacillus* were detected as transient strains at 16 and 21 d.a.g., respectively.

Root inoculation with *H. seropedicae* led to phylum-specific changes in the seed-borne endophytic community composition at the genus level. Regarding the phylum Proteobacteria, three new isolates belonging to the genera *Advenella*, *Brucella*, and *Brevundimonas* were detected in the seed-borne endophytic community isolated from inoculated roots, with the first two appearing 16 d.a.g. and the latter 21 d.a.g. Furthermore, a *Pantoea* strain previously not detected in the seed-borne endophytic community isolated from control roots was also detected 21 d.a.g. In addition, the actinobacterial genera detected in control roots were depleted in inoculated ones, where this phylum was represented by one strain belonging to the genus *Brevibacterium*, detected from 16 d.a.g. onward, and two strains, one persistent and the other transient appearing 21 d.a.g., from the genus *Microbacterium*. Regarding the Firmicutes phylum, the *Oceanobacillus* strain detected in control roots was not detected in inoculated ones, where the Firmicutes population was represented by two *Bacillus* strains, which were detected only 21 d.a.g. The inoculant *H. seropedicae* was not recovered from inoculated roots, indicating that although *H. seropedicae* persistence was not guaranteed over time, it still had an effect on the proliferation of the cultivable seed-borne endophytic community.

### Effects of *Herbaspirillum seropedicae* Inoculation on Root Biomass and on Seed-Borne Endophytic Community Growth

**FIGURE 2:**
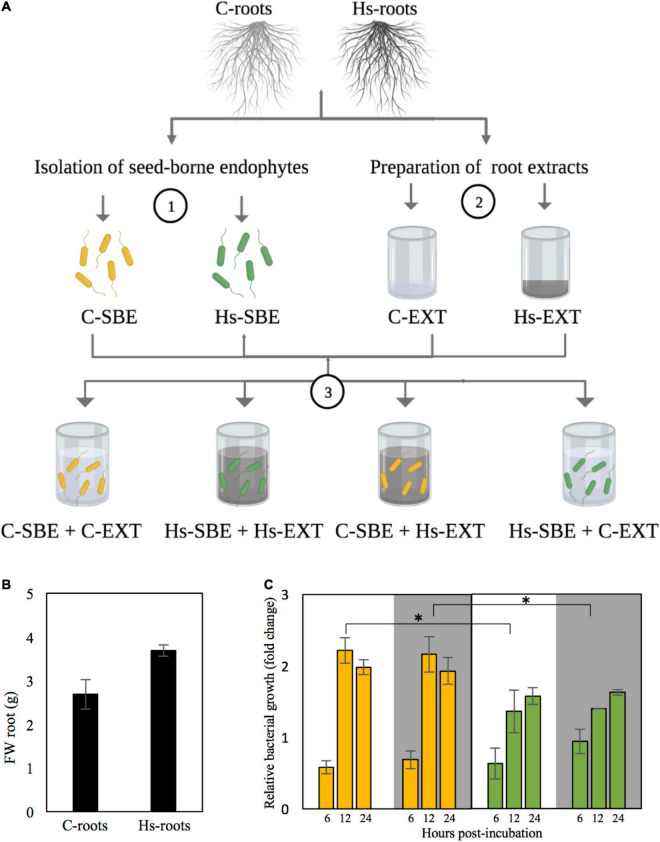
**(A)** Isolation of seed-borne endophytes from control and inoculated roots (depicted as yellow and green bacteria, respectively) (1), preparation of extracts of both inoculated and non-inoculated roots (depicted in white and gray, respectively) (2), and subsequent preparation of cultures containing each seed-borne endophytic community grown in extracts of either control or inoculated roots. **(B)** Root weight of both control and inoculated roots 21 days after germination (d.a.g). Asterisk indicates significant differences between control and inoculated roots (three biological replicates for each treatment, each consisting of a pool of 21 roots; mean ± SD, Student’s *t*-test for independent samples, *p* < 0.05). **(C)** Growth of seed-borne endophytes isolated from control and inoculated roots (yellow and green bars, respectively) in M9 supplemented with extracts of either control or inoculated roots (white and gray background, respectively) relative to their growth in M9 without extracts. For each timepoint, asterisks indicate significant differences between both communities when grown in extracts of either control or inoculated roots (Student’s *t*-test for independent variables; *n* = 3, mean ± SD; *p* < 0.05). C-roots, control roots; Hs-roots, inoculated roots; C-SBE, seed-borne endophytes isolated form control roots; Hs-SBE, seed-borne endophytes isolated form inoculated roots; C-EXT, extracts of control roots; Hs-EXT, extracts of inoculated roots. Created with BioRender.com.

To characterize the growth stimulatory effect of two different root endosphere environments (i.e., control roots and inoculated roots), both seed-borne endophytic communities isolated from control and inoculated roots were separately grown in M9 supplemented with the extracts of either control or inoculated roots, following the experimental procedure illustrated in Figure 2A. A prior determination of root fresh weight revealed that *H. seropedicae* inoculation stimulated root growth, increasing the biomass of inoculated roots by 25% compared with control ones (Figure 2B). Medium M9 supplemented with the extracts of either control or inoculated roots significantly stimulated the growth of both seed-borne endophytic communities relative to their growth in M9 medium in the absence of extracts (Figure 2C). Growth of the seed-borne endophytic community isolated from control roots in both extracts followed a similar trend without significant differences, and the same occurred for the endophytic community isolated from inoculated roots. However, independently of the origin of the extracts, the fold-change growth increase of the community isolated from control roots was significantly higher compared with the fold-change increase of the community isolated from inoculated roots 12 h post-incubation.

The different growth trends between both seed-borne endophytic communities, as well as the stimulatory effect of Hs on root growth, prompted us to explore the metabolic environment of both control and inoculated roots in the framework of Trp metabolism and its catabolic pathways leading to IAA production and other metabolites related to Trp catabolism.

### *Herbaspirillum seropedicae*- and Endophytic Community-Mediated Modulation of Tryptophan Metabolism in the Root Endosphere

**FIGURE 3:**
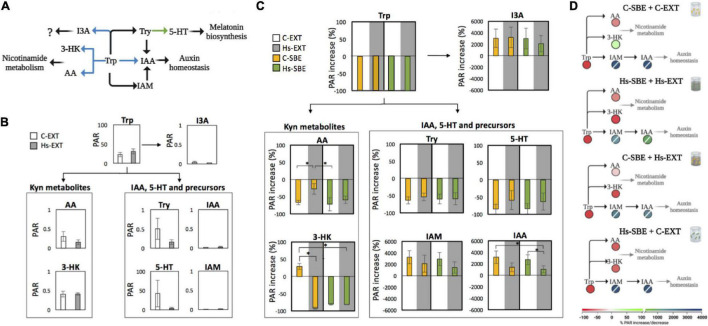
**(A)** Metabolic map showing analyzed Trp metabolites fitted into Trp catabolic pathways considered in this study; black arrows = Trp catabolic steps shared by bacteria and plants; blue arrows = steps exclusive of bacteria; green arrows = steps exclusive of plants (green arrows). **(B)** Peak area ratios (PAR) of detected Trp metabolites in extracts of control and inoculated roots (white and gray bars, respectively). **(C)** Percentage of increase/decrease of PAR values induced by seed-borne endophytic bacteria isolated from control and inoculated roots (yellow and green bars, respectively) grown in extracts of either control or inoculated roots (white and gray background, respectively) relative to PAR values of extracts of either control or inoculated roots without bacteria. According to comparisons made, two different statistical tests were carried out: when comparing both seed-borne endophytic communities grown either in extracts of roots from which they were isolated or in extracts from opposite treatment, asterisks indicate significant differences according to Student’s *t*-test for independent variables (*n* = 3, mean ± SD, *p* < 0.05). When comparing same community in different extracts, asterisks indicate significant differences according to Student’s *t*-test for dependent samples (*n* = 3, mean ± SD, *p* < 0.05). **(D)** Trp metabolites differentially altered by seed-borne endophytic communities when grown in extracts of either control or inoculated roots, fitted into Trp catabolic pathways considered in this study. Colors of circles represent% of increase/decrease for each metabolite. Transversal lines within circles indicate metabolites that were not detected in extracts of control roots nor in extracts of inoculated roots. Trp, tryptophan; Try, tryptamine; 3-HK, 3-hydroxykynurenine; AA, anthranilic acid, 5-HT, 5-hydroxytryptamine; IAM, indole-3-acetamide; IAA, indole-3-acetic acid; I3A, indole-3-carboxaldehyde (I3A); C-SBE, seed-borne endophytes isolated form control roots; Hs-SBE, seed-borne endophytes isolated from inoculated roots; C-EXT, extracts of control roots; Hs-EXT, extracts of inoculated roots. Created with BioRender.com.

The analysis of Trp-related metabolites carried out in this study was fitted into a metabolic map that included Trp catabolic steps that are both shared between bacteria and plants and exclusive to one of the two (Figure 3A). Overall, detected metabolites were Trp, Try, 3-hydroxykynurenine (3-HK), anthranilic acid (AA), 5-HT, IAM, indole-3-acetic acid (IAA), and I3A.

The extracts of both control and inoculated roots contained Trp, Try, 3-HK, AA, and 5-HT (Figure 3B). However, only slight differences between both treatments were observed, indicating that at the end of the experiment, *H. seropedicae* inoculation had minor effects on the levels of these metabolites in the root. In the case of 5-HT and Try, the differential levels of these metabolites could not be statistically confirmed due to the high variability among samples in the extracts of control roots.

In the second part of this assay, the potential of both seed-borne endophytic communities to alter Trp-related metabolites was estimated by measuring the% increase/decrease of each metabolite in the cultures containing each endophytic community grown in the extracts of either control or inoculated roots relative to the levels of the same metabolites determined in the extracts without bacteria (Figures 3C,D). Trp was depleted in all cultures. Among the IAA precursors analyzed, both communities decreased Try levels by 60%, independently of the extract in which they were grown. Furthermore, both communities increased dramatically the levels of both IAM and IAA when grown in the extracts of the roots from which they were isolated, with the community isolated from inoculated roots showing a significantly lower% of IAA increase. When grown in the extracts from the opposite treatment, this trend was reverted, with the community isolated from inoculated roots showing increased IAA levels compared with the one isolated from control roots, although this difference was not statistically significant. Further differences were observed for the Kyn downstream metabolites. When grown in the extracts of the roots from which each community was isolated, bacteria isolated from control roots increased 3-HK levels by 30%, whereas those isolated from inoculated roots decreased the 3-HK PAR value by 80%. However, when grown in the extracts from the opposite treatment, both communities behaved similarly, decreasing 3-HK levels by 80%. Regarding AA, its levels decreased by 70% when both communities were grown in the extracts of the roots from which they were isolated. However, a different trend was observed when grown in the extracts from the opposite treatment, with the community isolated from inoculated roots showing a significantly higher% of AA decrease compared with the one isolated from control roots.

Finally, I3A levels increased in both communities to a similar extent compared with IAA and IAM, independently of the extract in which they were grown.

## Discussion

### *Herbaspirillum seropedicae*-Mediated Modulation of Seed-Borne Endophytic Community Composition

The characterization of seed-borne endophytic communities performed in this work suggests that both plant developmental stage and *H. seropedicae* inoculation were two different driving forces shaping endophytic community composition. The evolution of both communities throughout root development revealed qualitative changes in community composition throughout plant age, where seed-borne endophytes isolated from inoculated roots showed a gradual increase in diversity at the genus level. Furthermore, both communities harbored both persistent and transient strains, sharing only one persistent strain of *Pantoea*. Representative members of this genus have been previously isolated from *T. aestivum* seeds, suggesting their long-term association with wheat plants (Kuźniar et al., 2020; Morales Moreira et al., 2021). This high persistence capacity also observed for *Curtobacterium* and *Microbacterium* in both control and inoculated roots, respectively, may enable members of the seed-borne endophytic community to rapidly multiply and increase their early abundance in the germinating seeds, which would, in turn, increase their legacy in posterior plant developmental stages. The rest of seed-borne endophytes isolated from both treatments were transient members of the community, suggesting that taxa that may lay outside of detectability thresholds in a given root developmental stage may later prove to be functionally important and increase their abundance, as previously postulated (Shade and Stopnisek, 2019).

Inoculation of *H. seropedicae* led to phylum-specific changes in seed-borne endophytic community composition at the genus level, which were manifested in all plant developmental stages. Inoculant-induced modulation of wheat indigenous actinobacterial populations was previously demonstrated using a mixed consortium as inoculum, where endophytic actinobacterial populations, including *Curtobacterium* and *Terrabacter*, were reduced by half (Conn and Franco, 2004). A similar trend was recently observed in resistant and susceptible wheat lines inoculated with the fungal pathogen *Tilletia controversa*, where the relative abundances of *Microbacterium* and *Curtobacterium*, among other actinobacterial genera, were differentially modulated (Xu et al., 2021). Inoculant-induced increase in the relative abundance of the genus *Brevibacterium*, observed in this study, has been demonstrated both through the addition of inoculants in compost and in healthy rhizospheric soils compared with diseased ones (Lee et al., 2021). However, this is the first evidence of the emergence of the genus *Brevibacterium* in wheat roots treated with a beneficial bacterium. Previous studies have shown plant growth-promoting attributes for this genus, such as phosphate solubilization, IAA and siderophore production, and alleviation of salinity stress in wheat plants (Ansari and Ahmad, 2019).

Regarding the Firmicutes phylum, inoculant-mediated shifts toward *Bacillus* spp. have been shown by Yin et al. (2013), which used *Pseudomonas fluorescens* 2P24 and CPF10 as inoculants. A mechanistic process explaining this late appearance, however, is lacking. A possible explanation could be based on the fact that *H. seropedicae* inoculation may lead to a phylum-specific modulation of spore-forming bacteria through the modulation of the root endosphere metabolic environment. Furthermore, no studies have focused on the modification of the relative abundances of the genus *Oceanobacillus* upon inoculant application, whereas the dynamics of *Oceanobacillus* populations have been mostly studied based on the combined effect of plant development and fertilization regimes (Chen et al., 2019). The increase in the diversity of Proteobacteria observed in inoculated roots is also consistent with previous studies. For example, a microbiome shift toward *Brucella* sp. was observed in tomato plants infected with *Ralstonia solanacearum* B3B through the inoculation of *Bacillus velezensis* B63 or *P. fluorescens* P142 (Elsayed et al., 2020). Both *Advenella* and *Brucella* genera have been included in the design of microbial synthetic communities able to yield consistent beneficial effects toward plants and have been shown to increase the number of lateral roots, to have ACC deaminase activity and phosphate solubilization (Tsolakidou et al., 2019).

Considering the absence of direct antagonism between all the members of the seed-borne endophytic communities isolated from control roots against those isolated from inoculated roots and between all isolated bacteria against *H. seropedicae*, the absence of certain genera such as *Curtobacterium*, *Oceanobacillus*, or *Terrabacter* in inoculated roots may be attributed to *H. seropedicae*-induced changes in the endosphere’s metabolic landscape, which may interfere with the proliferation of bacteria belonging to certain genera, while favoring other, previously unrepresented, bacterial members.

### Modulation of Tryptophan Metabolism Through an *Herbaspirillum seropedicae*-Mediated Change in Seed-Borne Endophytic Community Functionality

The potential of both seed-borne endophytic communities isolated from control and inoculated roots to modulate the endospheric levels of IAA and four IAA metabolic intermediates was investigated. Interestingly, the increase in IAA was differentially modulated according to the extract in which communities were grown. The visible reduction in IAA increase induced by the extracts of inoculated roots suggests the existence of an *H. seropedicae*-mediated modulatory signal that affects the potential of the endophytic community to accumulate or consume IAA, where the community isolated from inoculated roots may be better adapted to a higher IAA consumption. The results of this study suggest that *H. seropedicae* regulates seed-borne endophytic community functionality *via* changes in the endosphere metabolic landscape, which may, in turn, tip the metabolic balance toward an increased IAA turnover/degradation to produce a net positive effect on the host plant. This positive effect may be linked with the observed increase in root biomass. However, the consequences of *H. seropedicae*-mediated modulation of the seed-borne endophytic community remain elusive and need further experimental evidence to link a change in the endophytic community composition with root growth. In agreement with this hypothesis, previous studies have shown that bacterial degradation of IAA, and not only its production, is often required for full plant growth promotion and root development (Leveau and Lindow, 2005; Zúñiga et al., 2013). Moreover, although *H. seropedicae* is known to possess genes to produce IAA and can influence auxin-dependent gene expression in plants (Pankievicz et al., 2016), there is no clear mechanistic interpretation of growth promotion *via* inoculant direct IAA production in the endosphere.

To our surprise, *H. seropedicae* was not detected among the seed-borne endophytic community extracted from inoculated roots, or, at least, the inoculant was under the detectability thresholds according to the cultivable methods used in this study. This observation may be an indication that the beneficial effects of this inoculant do not necessarily comprise its presence throughout root development. Rather, our results point toward a microbiome modulation effect, where *H. seropedicae* effect may rely on a two-component mode of action, consisting of a shift in the endosphere metabolic landscape, which leads to a change in the seed-borne endophytic community composition and ultimately to the modulation of the endospheric IAA levels. Of four IAA precursors analyzed, the levels of only two of them, IAM and Try, were differentially regulated. In light of the results of this study, however, it is difficult to confirm whether the differences in IAA induced by seed-borne endophytic communities occur *via* the IAM pathway, the Try pathway, or both. The decrease in the levels of Try may also be attributed to the observed regulation of 5-HT levels, a derivative of Try where its role appears to be multifaceted, affecting plant growth while also facilitating cell–cell communication and quorum sensing (Knecht et al., 2016). As shown by the analysis of the extracts of both control and inoculated roots, inoculated roots may have reached an increased 5-HT turnover 21 d.a.g., as suggested by the lower PAR values in this treatment. In this context, root cells may benefit from intermediate metabolites of melatonin, such as 5-HT, that are modulated by seed-borne endophytes.

The alteration in the levels of the Kyn downstream metabolites measured in this study indicates an ulterior Trp-catabolizing pathway of both seed-borne endophytic communities isolated from control and inoculated roots in the endosphere environment. In the first place, an opposite modulation of 3-HK levels was observed between both communities when grown in the extracts of the roots from which they were isolated, which may be related to a change in the balance of the Kyn pathway of inoculated roots toward a higher 3-HK turnover. Furthermore, according to our observations, endophytic communities isolated from control and inoculated roots differentially consumed 3-HK and AA, respectively, depending on the extract in which were grown, suggesting that differences in the metabolic landscape of the endosphere can influence the modulation of certain molecules involved in Trp by seed-borne endophytes. Pieces of evidence supporting a microbial role in regulating the Kyn pathway belong mostly to clinical studies. For example, *Pseudomonas aeruginosa* can catabolize Trp through the Kyn pathway, interfering with the host immune response (Bortolotti et al., 2016). However, this is the first evidence of 3-HK differential modulation by seed-borne endophytes. Furthermore, AA is found in bacteria as both a precursor of Trp and as a product of Trp degradation. Interestingly, AA has also been proposed as a product of IAA degradation in different plant-associated bacterial species (Egebo et al., 1991). The differential regulation of the Kyn downstream metabolites may therefore have relevance not only for the NADP^+^/NADPH energetic balance but also for hormonal regulation within root tissues.

Finally, this is the first evidence of the production of I3A in the root endosphere by endophytic bacteria. This metabolite has been shown to be produced by diverse species from the *Lactobacillus* genus in the mammalian gut, being a hydrocarbon receptor with immunomodulatory functions (Zelante et al., 2013). The evidence that the levels of this metabolite are dramatically increased in the cultures containing seed-borne endophytic communities grown in the extracts of either control or inoculated roots highlights the importance of this Trp metabolite in plant–endophyte interactions. However, further studies are necessary to confirm the functions of this metabolite.

## Conclusion

In this study, we found some intriguing preliminary clues showing that root inoculation with the bacterial inoculant *H. seropedicae* strain RAM10 can shape the composition of the seed-borne endophytic bacterial community in the roots of wheat seedlings. These changes occur since early root developmental stages and are manifested along subsequent stages of the plant life cycle. Moreover, we demonstrate that these compositional changes can have profound impacts on the modulation of Trp metabolism in the endosphere and may potentially be a key mechanism of inoculant-mediated root growth promotion. Specifically, our results point toward a microbiome modulation effect, where the inoculant may rely on a two-component mode of action, consisting of a shift in the endosphere metabolic landscape, which leads to a change in the endophytic bacterial community composition and ultimately to the modulation of a series of Trp metabolites. In agreement with recent plant microbiome studies, the present work foresees the modulation of the seed-borne bacterial community as an important mode of action of microbial inoculants that is mediated both by the plant and its associated microbiota.

Future experimental approaches should focus on quantitative analyses of IAA and Kyn downstream metabolites in root extracts to understand the optimal levels of these compounds for full root growth promotion. In addition, members of endophytic bacterial communities should be individually screened in light of their capacity to modulate these metabolites to understand their single relevance for plant Trp metabolism and to determine how their functions can be shaped when grown in consortium with other bacteria. Moreover, culture-independent techniques could greatly help in the identification of non-detectable endophytes, which may be underrepresented yet have important roles in community functionality. Microbiome shifts require a better understanding of the communication occurring within the endophytic community, taking into account the role of the host metabolism as a key participant in the modulation of these processes. Multi-omics approaches, as well as biochemical studies on the activities of the enzymes involved in plant and bacterial Trp metabolism, will greatly help to understand both intra- and inter-kingdom interactions to open new horizons for plant metabolic engineering through a shift in its microbiota functionality.

## Data Availability Statement

The datasets presented in this study can be found in online repositories. The names of the repository/repositories and accession number(s) can be found in the article/Supplementary Material.

## Author Contributions

PC performed the experiments and data analysis and wrote the manuscript. JC played an integral part in the design of the experiments and data analyses regarding the microbiota isolation, characterization, and sequencing section, with SA contributing significantly to the laboratory work. GP and CS accompanied the methanolic extraction procedures and carried out the metabolomic analyses being an integral part of both the realization and discussion of this section. CC and RT were central parts for the design, supervision, and data analyses of all the sections of this manuscript. All authors contributed to the research and approved the final version of the manuscript.

## Conflict of Interest

The authors declare that the research was conducted in the absence of any commercial or financial relationships that could be construed as a potential conflict of interest.

## Publisher’s Note

All claims expressed in this article are solely those of the authors and do not necessarily represent those of their affiliated organizations, or those of the publisher, the editors and the reviewers. Any product that may be evaluated in this article, or claim that may be made by its manufacturer, is not guaranteed or endorsed by the publisher.
