# Supplementary material for: Modulation of the Wheat Seed-Borne Bacterial Community by Herbaspirillum seropedicae RAM10 and Its Potential Effects for Tryptophan Metabolism in the Root Endosphere
Source: Front Microbiol. 2021 Dec 23;12:792921. doi: 10.3389/fmicb.2021.792921 (PMC8733462; doi:10.3389/fmicb.2021.792921)
Supplement: Supplementary file 1 [file Table_1.DOCX]

**Table SM1.** Biochemial characterization and 16S-based identification (BLAST) of C-SEB and Hs-SEB.

| **ID** | **GRAM** | **KOH** | **CAT** | **OX** | **16S-based identification** | **query  length** | **% identity** |
| --- | --- | --- | --- | --- | --- | --- | --- |
| **C-SEB** | | | | | | | |
| **P1** | **-** | **-** | **+** | **+** | ***Pantoea* sp.** | **1125** | **98** |
| **P2** | **-** | **-** | **+** | **+** | ***Pantoea* sp.** | **1019** | **99** |
| **C1** | **+** | **+** | **+** | **+** | ***Curtobacterium* sp.** | **1078** | **99** |
| **C2** | **+** | **+** | **+** | **-** | ***Curtobacterium* sp.** | **892** | **100** |
| **C3** | **+** | **+** | **+** | **+** | ***Curtobacterium* sp.** | **1040** | **99** |
| **T** | **+** | **+** | **+** | **-** | ***Terrabacter* sp.** | **1091** | **99** |
| **Ob** | **+** | **-** | **+** | **-** | ***Oceanobacillus* sp.** | **1116** | **99** |
| **Hs-SEB** | | | | | | | |
| **P3** | **-** | **-** | **+** | **-** | ***Pantoea* sp.** | **1082** | **98.5** |
| **A** | **-** | **-** | **+** | **+** | ***Advenella* sp.** | **1196** | **99** |
| **O** | **-** | **-** | **+** | **+** | ***Ochrobactrum* sp.** | **1165** | **99** |
| **Br** | **-** | **-** | **-** | **+** | ***Brevundimonas* sp.** | **1168** | **99** |
| **B1** | **+** | **+** | **+** | **+** | ***Bacillus* sp.** | **1032** | **98** |
| **B2** | **+** | **+** | **+** | **-** | ***Bacillus* sp.** | **1131** | **99** |
| **M1** | **+** | **+** | **+** | **-** | ***Microbacterium* sp.** | **1103** | **99** |
| **M2** | **+** | **+** | **+** | **-** | ***Microbacterium* sp.** | **1134** | **99.4** |
| **Bv** | **+** | **+** | **+** | **+** | ***Brevibacterium* sp.** | **1136** | **99** |
