# Supplementary material for: Modulation of the Wheat Seed-Borne Bacterial Community by Herbaspirillum seropedicae RAM10 and Its Potential Effects for Tryptophan Metabolism in the Root Endosphere
Source: Front Microbiol. 2021 Dec 23;12:792921. doi: 10.3389/fmicb.2021.792921 (PMC8733462; doi:10.3389/fmicb.2021.792921)
Supplement: Supplementary file 2 [file Table_2.DOCX]

**Table SM2:** List of chromatographic retention time (RT), selected MRM parameters, declustering potential (DP), focusing potential (FP), entrance potential (EP), collision energy (CE), cell exit potential (CXP) for each measured analyte.

| Q1  (m/z) | Q3  (m/z) | RT  (min) | Analyte | DP | EP | CE | CXP |
| --- | --- | --- | --- | --- | --- | --- | --- |
| 225.0 | 110 | 2.6 | 3-hydroxy-kynurenine | 15 | 10 | 20 | 15 |
| 225.0 | 162 | 2.6 | 3-hydroxy-kynurenine | 15 | 10 | 20 | 15 |
| 228.2 | 210 | 2.6 | 3-hydroxy-kynurenine-^13^C_2_-^15^N | 15 | 10 | 20 | 15 |
| 228.2 | 110 | 2.6 | 3-hydroxy-kynurenine-^13^C_2_-^15^N | 15 | 10 | 20 | 15 |
| 177.0 | 160 | 5.7 | 5-hydroxytryptamine | 32 | 7 | 13 | 8 |
| 177.0 | 115 | 5.7 | 5-hydroxytryptamine | 32 | 7 | 45 | 14 |
| 209.0 | 192 | 5.5 | l-kynurenine | 33 | 4 | 13 | 10 |
| 209.0 | 94 | 5.5 | l-kynurenine | 33 | 4 | 20 | 14 |
| 215.2 | 169 | 5.3 | l-kynurenine D6 | 23 | 10 | 17 | 11 |
| 215.2 | 98 | 5.3 | l-kynurenine D6 | 23 | 10 | 17 | 11 |
| 154.0 | 136 | 6.5 | 3-hydroxy-anthranilic-acid | 40 | 10 | 18 | 20 |
| 154.0 | 80 | 6.5 | 3-hydroxy-anthranilic-acid | 40 | 10 | 40 | 12 |
| 157.1 | 139 | 6.4 | 3-hydroxy-anthranilic-acid-D3 | 15 | 10 | 15 | 8 |
| 157.1 | 83 | 6.4 | 3-hydroxy-anthranilic-acid-D3 | 15 | 10 | 35 | 4 |
| 205.1 | 146 | 7.5 | tryptophan | 30 | 10 | 30 | 15 |
| 205.1 | 188 | 7.5 | tryptophan | 30 | 10 | 30 | 15 |
| 161.0 | 144 | 8.1 | tryptamine | 36 | 10 | 29 | 7 |
| 161.0 | 117 | 8.1 | tryptamine | 36 | 10 | 31 | 7 |
| 138.1 | 120 | 9.4 | anthranilic-acid | 34 | 6 | 16 | 18 |
| 138.1 | 92 | 9.4 | anthranilic-acid | 34 | 6 | 32 | 13 |
| 142.2 | 124 | 9.3 | anthranilic-acid-D4 | 15 | 10 | 16 | 7 |
| 142.2 | 96 | 9.3 | anthranilic-acid-D4 | 15 | 10 | 30 | 9 |
| 175.1 | 130 | 9.8 | indole-3-acetamide | 19 | 10 | 16 | 9 |
| 175.1 | 158 | 9.8 | indole-3-acetamide | 19 | 10 | 25 | 9 |
| 183.1 | 136 | 10.8 | indole-3-acetic-acid D7 | 36 | 10 | 22 | 9 |
| 183.1 | 109 | 10.8 | indole-3-acetic-acid D7 | 36 | 10 | 43 | 6 |
| 176.1 | 130 | 10.9 | indole-3-acetic-acid | 40 | 10 | 23 | 10 |
| 176.1 | 103 | 10.9 | indole-3-acetic-acid | 40 | 10 | 36 | 10 |
| 146.1 | 118 | 10.8 | indole-3-carboxy-aldehyde | 48 | 10 | 25 | 7 |
| 146.1 | 91 | 10.8 | indole-3-carboxy-aldehyde | 48 | 10 | 28 | 7 |
| 157.2  157.2  160.2  160.2 | 130  117  118  130 | 15.4  15.4  14.3  14.3 | indole-3-acetonitrile  indole-3-acetonitrile  indole-3-acetaldehyde  indole-3-acetaldehyde | 30  30  33  33 | 10  10  10  10 | 30  30  20  36 | 15  15  8  8 |
