# Supplementary material for: Modulation of the Wheat Seed-Borne Bacterial Community by Herbaspirillum seropedicae RAM10 and Its Potential Effects for Tryptophan Metabolism in the Root Endosphere
Source: Front Microbiol. 2021 Dec 23;12:792921. doi: 10.3389/fmicb.2021.792921 (PMC8733462; doi:10.3389/fmicb.2021.792921)
Supplement: Supplementary file 3 [file Table_3.DOCX]

**Table SM3:** Chromatographic conditions.

| Time  (min) | Flow  (mL/min) | B (%) |
| --- | --- | --- |
| 0 | 0.300 | 0 |
| 11 | 0.300 | 40 |
| 13 | 0.300 | 95 |
| 21 | 0.300 | 95 |
| 22 | 0.300 | 0 |
| 33 | 0.300 | 0 |
|  |  |  |
